# The impact of loss of PEPFAR support on HIV services at health facilities in low-burden districts in Uganda

**DOI:** 10.1186/s12913-021-06316-4

**Published:** 2021-04-01

**Authors:** Henry Zakumumpa, Ligia Paina, Jess Wilhelm, Freddie Ssengooba, Eric Ssegujja, Moses Mukuru, Sara Bennett

**Affiliations:** 1 School of Public Health, Makerere University, Kampala, Uganda; 2 Bloomberg School of Public Health, Johns Hopkins University, Baltimore, USA

**Keywords:** Health systems, HIV, PEPFAR, Uganda, Health services, Donor transition

## Abstract

**Background:**

Although donor transitions from HIV programs are more frequent, little research exists seeking to understand the perceptions of patients and providers on this process. Between 2015 and 2017, PEPFAR implemented the ´geographic prioritization´ (GP) policy in Uganda whereby it shifted support from 734 ‘low-volume’ facilities and 10 districts with low HIV burden and intensified support in select facilities in high-burden districts. Our analysis intends to explore patient and provider perspectives on the impact of loss of PEPFAR support on HIV services in transitioned health facilities in Uganda.

**Methods:**

We report qualitative findings from a larger mixed-methods evaluation. Six facilities were purposefully selected as case studies seeking to ensure diversity in facility ownership, size, and geographic location. Five out of the six selected facilities had experienced transition. A total of 62 in-depth interviews were conducted in June 2017 (round 1) and November 2017 (round 2) with facility in-charges (*n* = 13), ART clinic managers (*n* = 12), representatives of PEPFAR implementing organizations (*n* = 14), district health managers (*n* = 23) and 12 patient focus group discussions (*n* = 72) to elicit perceived effects of transition on HIV service delivery. Data were analyzed using thematic analysis.

**Results:**

While core HIV services, such as testing and treatment, offered by case-study facilities prior to transition were sustained, patients and providers reported changes in the range of HIV services offered and a decline in the quality of HIV services offered post-transition. Specifically, in some facilities we found that specialized pediatric HIV services ceased, free HIV testing services stopped, nutrition support to HIV clients ended and the ‘mentor mother’ ART adherence support mechanism was discontinued. Patients at three ART-providing facilities reported that HIV service provision had become less patient-centred compared to the pre-transition period. Patients at some facilities perceived waiting times at clinics to have become longer, stock-outs of anti-retroviral medicines to have been more frequent and out-of-pocket expenditure to have increased post-transition.

**Conclusions:**

Participants perceived transition to have had the effect of narrowing the scope and quality of HIV services offered by case-study facilities due to a reduction in HIV funding as well as the loss of the additional personnel previously hired by the PEPFAR implementing organizations for HIV programming. Replacing the HIV programming gap left by PEPFAR in transition districts with Uganda government services is critical to the attainment of 90–90-90 targets in Uganda.

**Supplementary Information:**

The online version contains supplementary material available at 10.1186/s12913-021-06316-4.

## Background

Sub-Saharan Africa (SSA) is the region with the highest HIV burden in the world [1]. Out of the 37 million living with HIV globally, 26 million live in Sub-Saharan Africa (SSA) [2]. In addition to having a high HIV burden, this region is characterized by weak health systems and an under-investment in the health sector [3]. As such, many SSA countries depend substantially on international assistance for their national HIV responses [4]. The President’s Emergency Plan for AIDS Relief (PEPFAR) and The Global Fund to fight AIDS, Tuberculosis and Malaria have been primary sources of support [4]. Indeed, donor support has enabled SSA to register important strides in the quest to attain UNAIDS’ 90–90-90 targets for global HIV epidemic control by ensuring that 90% of persons living with HIV (PLWH) know their diagnosis, 90% of diagnosed PLWH are enrolled on antiretroviral therapy (ART) and 90% of PLWH on ART should be virally suppressed by 2020 [5].

Over the past decade, there have been increasing donor transitions away from middle-income countries as they ‘graduate’ from donor support for HIV [6–9]. To date, research has focused on PEPFAR and Global Fund transitions away from middle-income countries such as Thailand, Vietnam and South Africa [10–13]. Because low-income countries have not previously been as affected by donor transition as middle-income countries [6], there has been little research exploring the impact of loss of donor support on HIV services in resource-limited settings [6], and data on the perspectives of local stakeholders in this regard are even sparser [13]. Previous studies have documented the effects of loss of donor support on HIV services in India [14, 15], the Caribbean [10], Peru [16] and South Africa [11, 12]. Research around donor transitions is vital to understanding the dynamics, processes and challenges involved in these transitions and is critical in informing decision making by donors as well as in devising responses by donor-recipient governments [6, 9, 10]. This is especially important in the quest to sustain the gains registered in HIV epidemic control in low and middle-income countries, as a result of more than $ 500 billion in investment over the past two decades [1, 8].

PEPFAR announced a new strategy known as ‘PEPFAR 3.0’ for the period 2013–2019 which aimed at ensuring better efficiency and sustainability in its support of national HIV responses in its 15 focus-countries [10, 17]. As part of this broader strategy, PEPFAR announced the ‘geographic prioritization’ policy, which sought to align aid with the sub-national level disease burden within countries supported by PEPFAR [17]. To improve the allocative efficiency of its HIV support, PEPFAR sought to move away from generalized national responses to a more targeted approach, ‘pivoting’ towards sub-regions with higher HIV incidence [10]. Under this strategy, sub-regions with relatively high HIV burden were to receive increased funding while ‘low yield’ regions or those with relatively low HIV burden would receive significantly less support [17].

In Uganda, PEPFAR implemented the geographic prioritization (GP) policy between 2015 and 2017 [18]. Under the GP policy, 734 ‘low volume’ health facilities were designated to lose the support of implementing organizations (IPs) countrywide. A total of 94 PEPFAR-supported facilities based in 10 low-burden districts were meant to transition to Ugandan government support [18]. The remaining facilities designated for transition consisted of ‘low volume’ sites that did not report sufficient numbers of HIV+ individuals tested and treated. PEPFAR planned to maintain support for 419 facilities in districts with moderate HIV burden known as ‘maintenance’ and intensified support in 1384 health facilities in ‘scale-up’ districts with a higher HIV burden [19, 20].

Due to the high level of dependence on PEPFAR support for HIV services, any changes in the scale of donor aid received would potentially impact HIV service delivery in Uganda. Although previous studies in Uganda have utilized quantitative approaches to assess the impact of PEPFAR support on health systems [21, 22], the perspectives of primary beneficiaries and frontline health workers have received relatively little empirical attention to date [13]. The objective of this study was to explore the perspectives of patients and HIV service managers on the effects of loss of PEPFAR support on HIV service delivery in Uganda.

## Methods

### Research design

This was a qualitative study of health facility cases nested within a broader mixed-methods evaluation of the effects of implementation of PEPFAR’s geographic prioritization policy on HIV and non-HIV services in Uganda and Kenya [18, 23]. The study used a case-study design which is recommended for in-depth investigation of complex phenomena within organizations [24, 25]. More specifically, the case studies aimed to understand the perspectives of varied, local-level stakeholders in Uganda on how transition had affected HIV and non-HIV service delivery.

### Study sites and sample selection

Six health facilities that received PEPFAR support between 2012 and 2014 were purposively selected (Table 1**).** The sample of health facilities was intended to reflect diversity with regards to level of care in the Ugandan health system (primary, secondary), ownership-type (private/public) and transition status (Central support/ maintenance). The majority of included facilities represented the public sector, which predominates in rural areas, and those transitioning to central support, as this is the phenomenon which we were primarily interested in.

**Table 1:** The six case-study facilities and their characteristics

	PUB-001	PUB-002	PUB-003	PUB-004	PFP-001	PNFP-001
**Ownership-type**	Public	Public	Public	Public	For-profit	Not for profit
**Level of care**	General public Hospital	Health Centre IV	Health Centre IV	Health Centre IV	Health Centre II	Health Centre III
**Setting**	Urban	Peri-urban	Peri-urban	Rural	Urban	Rural
**Transition status**	Central support	Central support	Central support	Maintenance	Central support	Maintenance
**HIV services offered post-transition**	VCT, ART, PMCT	VCT, ART PMCT	VCT, ART,PMCT	VCT, ART,PMCT	VCT	VCT
**Year of transition**	2015	2017	2016	2017	2016	2016
**Cumulative number on ART (Jan-2016)**	3189	2193	778	776	ART services not provided.	ART services not provided.

*PMTCT* prevention of mother to child transmission, *VCT* voluntary counselling and testing of HIV

For each case study facility, respondents were purposively selected from among facility personnel, as well as district staff including members of the District Health Team (such as the District Health Officer (DHO), District HIV focal person) and representatives of the local PEPFAR implementing organization. ART patients were selected from four ART-providing facilities for focus group discussions (FGDs) (Table 1), on an ART clinic day with the assistance of the in-charge. Selected patients had been enrolled in care during the period when the health facility benefited from PEPFAR funding and so we could elicit comparative perspectives on HIV services before and after the transition period.

### Data collection

Data were collected over two rounds in May 2017 (round 1, just after transition) and six months later, in November 2017 (round 2) to enable an understanding of the longer-term effects of donor transition on HIV services in the case-study facilities, and in particular, potential processes of adaptation over time.

#### In-depth interviews

Sixty-two semi-structured In-depth Interviews (IDIs) were conducted (Table 2**)**, with 31 IDIs in each of the two rounds of data collection (supplementary file). Where possible we purposively sought to interview the same respondents in each round. IDIs were conducted with facility in-charges (*n* = 13) and ART clinic in-charges in the six health facilities (*n* = 12) to explore their perceptions of the changes in health-system components and HIV service delivery resulting from transition. Examples of the questions posed include 1) *What kind of support were you receiving from PEPFAR before transition? 2) Before PEPFAR support ended what kind of help did you get to enable you get ready for the end of that support?* (**Supplementary file 1).** Twenty-three IDIs were also conducted with district health managers (*n* = 7) and representatives of local PEPFAR implementing organizations (*n* = 14). The interviews were conducted in English by HZ and ES assisted by four Research Assistants (RAs). These face-to-face interviews were held on-site in interviewees’ offices at the case-study facilities.

**Table 2:** Category of respondents for case-study

Respondent type	Round 1	Round 2	Total
Facility in-charges	7	6	13
ART clinic in-charges	6	6	12
District Health Team leaders	12	11	23
Representatives of PEPFAR Implementing Organizations	9	5	14
**Focus Group Discussions**	6	9	15
Number of participants	38	34	72

#### Focus group discussions (FGDs)

A total of fifteen FGDs were conducted between April and June 2017 (six in round 1), and in November 2017 (nine in round 2) by HZ and ES with the help of two Research Assistants experienced in qualitative research. The FGDs sought to understand the perspectives of patients on changes in HIV service delivery at case-study facilities post-transition. The questions posed in the FGDs are attached (Supplementary File 2). The FGDs were gender-segregated and conducted in English. In all, a total of 72 patients participated in the focus group discussions. The FGDs were conducted on-site at the case-study facilities on an ART clinic day after patients enrolled on ART had completed their scheduled monthly review sessions. It is worth noting that in several cases, the clinics proposed that ‘expert patients’ participate as they were considered a rich source of information both as patients and as individuals involved in the running of ART clinics. ‘Expert patients’ are regular patients seeking HIV care who are selected to play supportive roles at HIV clinics (such as in managing triage systems). A select number of them earned a wage income for their supportive roles. On average, FGDs lasted between 45 and 60 min.

### Data analysis

Interviews were audio-recorded and transcribed verbatim by five Research Assistants and translated into English (when needed). The transcripts were subsequently uploaded into Atlas.ti for data management.

We followed the procedures recommended for qualitative data analysis by Miles & Huberman (1994) [26]. To this end, data were analyzed in an iterative process involving four major stages; 1) Data familiarization through multiple readings of interview transcripts 2) Evolving a code framework derived from an inductive approach based on the data 3) Abstraction of the coded data into thematic matrices and 4) Overall interpretation and synthesis [26].

Data analysis was carried out in two phases. We first analyzed data collected during the first round of interviews and focus groups conducted in May 2017, this informed revisions to interview guides for the second round of data collection in November 2017. The second round of questions were more focused on how respondents’ experiences changed over time. The team wrote up case-studies for each facility during each round, in order to inform a holistic perspective of what was happening at each facility over time. Finally, we conducted a cross-case analysis exploring patterns across facilities and the extent to which similar versus different themes arose.

## Results

The findings emerging from this study are presented in two parts. In the first part we present findings relating to changes in the range of HIV services offered post-transition in case-study facilities. In the second part we present patients’ and HIV service managers’ perceptions of changes in the quality of HIV services post-transition.

### Changes in the range of HIV services offered

While core HIV services (e.g. testing, treatment) by case-study facilities which were offered prior to transition were sustained, we found that more specialist services as well as various supportive services offered by these facilities often ceased. The reported ‘narrowing’ in the HIV services offered included the loss of specialized pediatric HIV services, the discontinuation of “mentor mother” programs, the winding down of nutrition support to HIV clients and the end of free HIV testing services at a for-profit clinic.

#### Specialized pediatric HIV services ceased

Our focus groups (FGDs) with patients revealed that specialized pediatric ART services ceased at select case-study facilities (PUB-002, PUB-003, and PUB-004). Prior to transition, facilities providing ART services separated pediatric clients from adults on ART clinic days. During on-site visits to PUB-002 and PUB-003 by investigators, it was observed that children were mixed with adults in the patients’ waiting area and in the queue to see clinicians. This was confirmed in interviews with informants.

‘We used to have specialized HIV services for teenagers and adolescents but now we incorporate them with the adult services because the IP (implementing organization) closed’ [ART clinic in-charge, PUB-003]

Interviewees reported that the PEPFAR implementing organizations provided incentives to care takers of pediatric patients to improve their adherence by providing them with transport money for attending appointments for review as well as receiving nutrition support such as milk and soya all of which were not sustained post-transition.

#### Discontinued ‘mentor mother’ adherence support program

The ‘mentor mother’ model was discontinued at PUB 002 and PUB 003. ‘Mentor mothers’ were model female HIV clients who were recruited as informal health workers and paid a monthly allowance to offer adherence support to care-takers of children enrolled on ART as well HIV positive expectant mothers for prevention of mother to child transmission (PMTCT). Mentor mothers traced clients who were lost-to- follow-up within their communities. Monetary allowances to ‘mentor mothers’ were paid directly by the PEPFAR implementing organization. Once the payment of allowances ceased, ‘mentor mothers’ could no longer afford to spend prolonged periods at ART clinics providing adherence support.

‘These mentor mothers some of them stopped coming. We could not do follow-ups so it created a big gap in our services ‘[Health worker, PUB-002]*.*

‘Mentor mothers were attached to pregnant mothers and children below 18 months. They would counsel them, make sure they take their medication and encourage them with counselling. Clients would come [to the clinic] because of their love and care’ [ART clinic in-charge, PUB-003]

The loss of funding for the ‘mentor mother’ program at the facility was described as a loss of a vital link to the community because it undermined the capacity of health facilities to do client follow-up and adherence support for their pediatric and adolescent clients.

‘The challenge came with mentor mothers. The mothers would do follow-up and that’s why you find that some children, their viral loads have not been suppressed. Once they disappear they take time to come back’ [Health worker, PUB-002]*.*

#### Loss of nutrition support to HIV clients

From the focus group discussions (FGDs) with patients at three sub-district health centres (PUB-002 PUB-003, PUB-004) it emerged that the nutrition support provided to patients during ART clinic days was discontinued after donor transition. It was reported that nutrition support was part of the core package of care offered by implementing organizations to health facilities in their regions of operation. Nutrition support was thought by respondents to be critical due to ART medication which increased appetite, and the absence of adequate food, which led to severe hunger in patients. To enhance adherence to treatment, food support was provided such as maize porridge served to clients attending the ART clinic as well as maize flour provided to patients for consumption at home.

‘The implementing partner used to send the sick very many things. It used to send us milk, baby soya and other things which we do not see any more’ [Patient FGD, PUB-002]*.*

Most of the case-study facilities (such as PUB 002) were based in areas which were prone to food insecurity and catered to the majority rural poor who live off the land. Basic meals were not a guarantee in these settings and yet adherence to ART demanded regular food in-take.

‘We are facing famine in this area. You see a client has lost 10 kilograms. We used to have nutrition support. We used to have a nutritionist from within the hospital. Clients could get food from here. This was affected by transition’ [Patient FGD, PUB-002]

#### Free HIV testing services ended at a for-profit clinic

In-depth interviews with health workers and the in-charge of a case-study for-profit clinic (PFP-001) indicated that PEPFAR implementing organizations provided funding that enabled them to provide free HIV testing services for the densely populated slum area surrounding the clinic. The support included the provision of free HIV test kits (and related commodities) as well as monetary allowances to clinic staff during field outreaches for demand-creation campaigns among most-at-risk populations such as sex workers and *boda boda* (motor cycle taxi) riders living within the community.

‘The implementing organization used to support us in HIV Counseling and Testing (HTC) but that ended in 2015. They provided us with inputs necessary for HTC services such as HIV test kits and laboratory reagents, monetary allowances for our staff engaged in project activities such as outreaches. [Facility In-charge, PFP-001]

The loss of PEPFAR support was reported to have had far-reaching effects on HIV testing rates at the clinic (PFP-001) which was compelled to introduce a fee for HIV tests. Both health workers and patients concurred that HIV testing rates had declined significantly after transition. Besides the effects of introducing a fee for paying for routine HIV tests at PF-001, HIV testing rates were also reported to have declined remarkably post-transition due to the discontinuation of community-based outreach activities for HIV testing which had been key in combating the stigma surrounding HIV testing.

‘During the time (PEPFAR Implementing organization) supported us, our HIV testing services were free of charge. In a day we could get 400 or 500 clients. On peak days, we could even get 1,000 clients. Now they are down to 100’ [In-charge, PFP-001]*.*

‘I used to look for the people (community outreach). Now I wait for the people. But how many people come here? They hardly turn up. They only do HIV tests when they have come for malaria treatment or some other ailment. We only convince those who have come to the clinic to do HIV tests. There is a very, very big difference between now and the time we had PEPFAR support. The attendance these days is very poor’. [Health worker, PFP-001]*.*

The sense that the loss of PEPFAR support through transition had contributed considerably to a decline in HIV testing rates at this private-for-profit facility was corroborated by representatives at the district level:

‘They were affected (PFP-001). The turn up at the clinic reduces, the total number of clients they serve reduces and they cannot do outreaches anymore yet VCT (voluntary counselling and testing) is done at outreach posts because it is difficult for someone to wake up and go to a clinic for an (HIV) test but when they find you (clients) in your comfort zone chances of getting tested are higher’ [Representative of PEPFAR Implementing organization, PFP-001]

### Perceptions of change in the quality of HIV services offered

Patients reported changes in the quality of HIV care offered in some case-study facilities following transition. In comparing the focus group discussions held in May 2017 (round 1) and those conducted six months later in November 2017, patients at four of the six case-study facilities described significant variations in the quality of HIV services offered at PUB-01, PUB-02, PUB-03 and PUB-04 when compared to the pre-transition phase. Both patients’ and health workers’ perceived a decline in the quality of HIV services post-transition. The specific aspects cited include the notion that ART medicines stock-outs had become more frequent (PUB-02, PUB-03, PUB-04), perceived increases in out-of-pocket expenditures on HIV care (PFP-01, PUB-03) a reduction in basic supplies and commodities for handling patients and a decline in patient-centric and holistic HIV care (PUB-02, PUB-03, PUB-04).

#### Perceived changes in patient-centered HIV care

In the focus groups, patients described enduring longer waiting times in the second round compared to the previous one due to what was described as a less efficient patient flow system post-transition which, in part, was attributed to a reduction in staffing especially the loss of the supportive roles played by ‘expert patients’ in managing triage systems at ART-providing facilities (PUB-002, PUB-003). Patient flow management in ART clinics at public facilities (PUB-002, PUB-003) was described as less organized compared to the pre-transition period and that the patient waiting area did not have a tent or a protective shade in the patient waiting areas. Patients were unequivocal in relaying the notion of a change in the general quality of HIV care.

‘The implementing partner (IP) used to provide patient files but we don’t have them now. So (PUB-002) has to buy them so that our information is organized. The IP would support sweeping of the rooms where we sit, provide these dusters, provide these seats because (PUB-002) hadn’t bought theirs so now that is the gap. We used to sit under the IP’s tent because (PUB-002) doesn’t have any so we sit in the corridor, we sit under the hot sun’ [Patient FGD, PUB-002]*.*

The sense that the quality of HIV services had changed post-transition was a perception held not only by patients but across a broad spectrum of informants including the health workers themselves.

‘Services have continued but at a little lower level than they would have if support had continued. The way we do things is not the way we used to do things. The implementing organization had high quality, but now?’ [Health worker, PUB-002]*.*

‘The implementing partner helped us to bring services to a different level and I know as time goes on staff will decline and services will go down’ [Facility in-charge, PUB-003]*.*

#### More frequent stock outs of antiretroviral medicines

Across four case-study facilities, based on interviews with facility personnel and focus groups with patients, it emerged that stock outs of anti-retroviral medicines had become more frequent compared to the pre-transition period (PUB-001; PUB-002; PUB-003 and PUB-004).

‘Sometimes we get stock outs of ARVs especially second-line [regimens]. This this did not happen because back in the days of [name of implementing organization] stock was always provided on time’ [Patient FGD, PUB-001]

The shortage of pediatric ARV drugs was also frequently cited in three (of the six) case-study facilities (PUB-002, PUB-003-PUB-004) as was the shortage of septrin (cotrimaxazole).

‘When we were still with those people [name of implementing organization], they would provide ARVs for children but now it’s out of stock’ Facility in-charge, PUB-002*.*

When we probed interviewees on why stock-outs had become more frequent after transition, the District Health Officers and facility in-charges described the kind of support PEPFAR implementing organizations (IPs) offered in managing HIV commodities supply chains. This included assigning IP personnel to the task of ensuring sufficient stock of HIV commodities across all health facilities within sub-regions under their purview. The IPs maintained emergency stocks of commodities, which they drew upon during stock-out events. IP staff were also instrumental in redistributing HIV commodities across health facilities such as taking excess stock from one health facility and distributing it to another experiencing shortage. These efforts were supported with a dedicated transport budget for fuel and vehicles for the purpose.

‘The implementing partner used to support us to re-distribute some of these supplies from other districts where they had excess’ [District Health Team leader, PUB-002]*.*

‘The IP would come in during emergencies of stock outs. They would fill gaps when NMS (National Medical Stores) supplied less stock than we needed and they would fill the gap in between the NMS supply cycle’ [ART clinic in-charge, PUB-001]*.*

The additional personnel recruited by PEPFAR to manage HIV commodities supply chains and the transport costs provided for stock re-distribution by PEPFAR IPs were support mechanisms that were not sustained by the case-study health facilities after transition. Although ordinarily districts in Uganda retain overall responsibility for social services provision, interviewees widely perceived districts as cash-strapped and pre-occupied with basic functionality as sub-national units. This was especially the case for new districts, recently formed through the government’s rapid decentralization efforts. The majority of facilities in our sample were located in districts created after 2014, and therefore several of them experienced challenges related to the establishment of new districts.

‘We have been supporting the district to play its role. But even the district has challenges. It’s a new district and still has some challenges to overcome. We support the district to do routine supervision of the health sector. They need fuel. You have to send a car with fuel and a driver to move them about’ [Representative, implementing organization, PUB-002]

‘HIV services cannot work without donor support. The district has no funding to support HIV activities. When you look at the budget of the district, it is very small. It cannot support HIV activities.’ [District Health Team member, PUB-003]

From the interviews with facility personnel and representatives of PEPFAR implementing organizations it emerged that districts did not provide any significant funding to replace PEPFAR support post-transition although this was the anticipated action for districts following transition.

‘The facility budget has not been adjusted to respond to the problems (transition). Even just allocating one million shillings ($270) in a quarter can do something but that hasn’t happened so they just left them there (PUB-001)’ [Representative, PEPFAR implementing organization, PUB-003]*.*

‘I don’t know how to describe our district but our district believes health is well supported and they don’t put any money in health at all. That has been my major quarrel with them. Whatever is got from (district) revenue is used for other things’ [Facility in-charge, PUB-002]*.*

Of the six districts, one district was reported to have stepped up when the PEPFAR contract with the implementing organization supporting PUB-003 elapsed. This relatively well-established district in Eastern Uganda (formed prior to district splitting in 2014) provided fuel for transporting laboratory samples from the ART clinic to a regional lab hub in the region as well as becoming more engaged in on-site supervision at PUB-003.

#### Transition effects on basic supplies and commodities

Health workers associated the loss of PEPFAR support with a reduction in the stock of basic supplies available for routine service delivery. The shortage of syringes and gloves was frequently mentioned by health workers across case-study facilities.

‘We used to be supplied with things like gloves and syringes and if there were no syringes in OPD (outpatients’ department) or maternity, we would supply those units with syringes and gloves. But right now the patient can even convulse to death without a syringe’ [Health worker, PUB-002]*.*

It emerged from the interviews with facility personnel that the PEPFAR implementing organizations periodically supplied health facilities with basic supplies (such as gloves and syringes). Although these were primarily meant for HIV services such as in the case of commodities for HIV testing, health workers frequently utilized these same supplies for routine non-HIV services owing to the resource-limited settings in which they operated.

‘We were receiving supplies for SMC (Safe Male Circumcision). A lot of them …we could use the excess of these supplies in other departments. This facility used not to lack gloves. Because in the (HIV test) kits there were excess gloves. So we would give then to other departments which wanted them. Cotton and gauze would go to maternity (section)’ [ART clinic in-charge, PUB-003].

## Discussion

Although donor transition from HIV programs is increasingly common, there has been limited empirical attention accorded to examining its effects on HIV service delivery from the perspective of patients and HIV service managers. We sought to understand local stakeholder perspectives on the early effects of loss of PEPFAR support on HIV services in transitioned facilities in Uganda. Overall, participants perceived transition to have had important impacts on HIV service delivery in transitioned health facilities. Participants described transitioned facilities and districts as having been unable to replace lost PEPFAR investments in HIV programming and the requisite management systems for the HIV response in their districts. As a result, there was wide acknowledgment from participants that although core HIV services had been maintained, more specialized services and support services had in some cases been ceased.

While patients and HIV service managers perceived HIV services to have been noticeably affected by the loss of PEPFAR support, findings from the broader study that included a health facility survey and analysis of health management information system data, concluded that ‘there is insufficient evidence to suggest negative impacts on volume of HIV services’ post-transition [18, 23]. Another sub-analysis of the larger study showed that PEPFAR transition had some positive impacts such as the increased integration of HIV with non-HIV services in transition facilities, as well as providers devising alternative funding sources for HIV services no longer supported by PEPFAR [20]. One of the challenges then for this study is to reconcile the perspectives of our respondents on transition (which were largely negative) with the data collected from other components of the study. While to some extent respondent perspectives may be biased by the direct personal impacts of transition (such as loss of allowances for expert patients) we believe, as outlined below, that there are additional explanatory factors that need to be taken into account.

### A narrowing in the range of HIV services offered post-transition

Our study adds evidence from a low-income country to the sparse but growing body of evidence reporting a narrowing in the range of HIV services offered following donor transition most of which has emerged out of middle-income countries [27]. Rodriguez and colleagues [15] in a case-study of health facilities in India which received support under the ‘Avahan initiative’ describe participants’ perceptions of a narrowing in the scope of HIV services offered by providers following loss of donor HIV support for community HIV outreach activities. A USAID study published in 2017 found ‘an inability to sustain the compendium of services in the post-transition period’ for countries in middle-income countries undergoing donor HIV transitions [28].

Participants in this study reported that the cessation of the ‘mentor mother’ pediatric adherence support mechanism at case-study facilities had contributed to declining viral suppression among pediatric patients. Studies in Uganda suggest that viral suppression among pediatric patients is as low at 23% [29] and with the loss of tailored programs for pediatric patients, it is possible that even greater challenges to viral suppression among this group may be seen.

In this study we found that free HIV testing services were discontinued in some facilities. Additionally, community HIV testing outreaches ceased post-transition and the Uganda government had not stepped in to fill the void. It is important to note that developing and implementing cost-effective strategies to sustain community engagement and patient-centered services takes time and effort, and will likely need careful planning in sub-national units affected by donor transition. Previous studies have highlighted the importance of community HIV outreach activities in promoting linkage to HIV care [30, 31].

### Declining quality of HIV services following donor transition

In this study, patients and health workers perceived a decline in the quality of HIV services offered at case-study facilities following loss of PEPFAR support. In a study in South Africa by Katz and colleagues [32], patients reported that HIV services in the post-transition period were less patient-centred and ‘largely focused on dispensing medication and on throughput, rather than holistic care’. A study by Bennett and colleagues [14] in India found that 30% of providers who received Gates Foundation support for a large HIV prevention program reported stock outs of commodities such as condoms and lubricants a few months after transition to the government of India. A study in Nigeria by Banigbe and colleagues [33] assessed the effects of a policy shift by PEPFAR towards increasing country ownership of HIV services instituted in October 2014. This policy shift entailed funding cuts amounting to $ 83 million. This study found that the implementation of this policy resulted in compromised quality of care. More specifically, ‘service delivery was hampered by interrupted laboratory services and reduced wages and staff positions leading to reduced provider morale, and compromised quality of care’ [33, 34].

### Transition effects on health-system components

Our analysis of local stakeholder perspectives suggests that PEPFAR transition had impacts on health-system components in case-study districts [35]. The loss of the additional health workforce recruited by PEPFAR implementing organizations for HIV programming at the regional-level and at the facility-level impacted HIV service delivery. Specifically, the extra specialist staff recruited for HIV programming at the sub-national level for instance to strengthen ART pharmaceutical supply chains and for HIV services quality monitoring was lost post-transition. These newly created specialist positions at the regional-level were not provided for in the established Ugandan public service structure and, therefore, these cadres could not be absorbed onto the public payroll post-transition. At the facility-level, informal workers such as ‘mentor mothers’, ‘expert patients’ and ‘community linkage facilitators’ filled critical staffing gaps common at the frontline level of service delivery in Uganda [36]. The loss of this extra personnel had far-reaching effects. Specifically, we found that the loss of field outreach allowances for facility personnel and ‘community linkage facilitators’ resulted in reduced community outreach activities which depressed demand for facility-based HIV testing services. The loss of PEPFAR implementing organization support in managing ART supply chains was perceived to have contributed to the increased frequency of antiretroviral medicines stock-outs. Our study adds to the literature highlighting the influence of human resources for health on service delivery and broadly on the notion of dynamic interactions in health system sub-components [37, 38]. We also observe that some of the effects caused by this loss of staff may be lagged, meaning that impacts on patient service data may take a longer period of time to occur.

With regard to leadership and governance, the majority of case study facilities (5 out of 6) hailed from new districts created after 2014 [39], which had narrow resource envelopes and weak leadership and management capacities for filling the HIV funding and programming gap left by PEPFAR [40, 41]. In Uganda, there exists a heavy dependence by sub-national units on central government grants for even basic operational funds. Without central government budget support to local governments, districts are scarcely able to mount effective responses to donor transitions on their own [39]. Broader governance deficits and the weak financial capacity in transition districts rendered them unable to fill the void in HIV programming post-transition [42]. Previous studies have highlighted the challenges of realizing the full potential of decentralization in improving health services delivery [43, 44].

### Study implications for donors and recipient governments

Our study found that although PEPFAR intended to transition low HIV burden regions to local ownership, there was no substantial response by the Uganda government in replacing the HIV financing and programming gap left by PEPFAR in regions affected by the GP policy. Burrows and colleagues [6] have called for civil society activism to catalyze appropriate responses from donor-recipient governments during post-transition phases. Sub-national government units in our study appeared to be unprepared to effectively respond to PEPFAR transition and we did not hear of a significant civil society presence at the sub-national level. One strategy to better support sub-national actors to respond to donor transition is outlined by Amaya and colleagues [16], who have called for donors to align their transition road maps with host country budget cycles and policies. Additionally, PEPFAR and other donors undergoing transition can consider a sparse but growing literature proposing ‘best practices’ in donor transitions [6, 9], including from Vogus and Graff [10], who recommend six transition strategies that include the need to communicate early on transition intentions, jointly developing transition road maps and aligning with country context and policy frameworks. Based on our research, we recommend that particularly the communication and alignment takes place not only at the national, but also at sub-national levels.

**FIG. 1:**
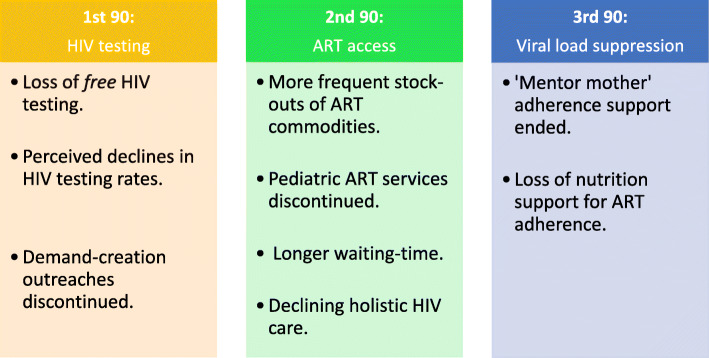
Visual illustration of impact of pepfar transition on UNAIDS 90–90-90 Targets

With regard to the Uganda government, our study demonstrates an urgent need for increasing domestic financial responsibility in replacing lost PEPFAR support in the 10 transition districts and the 734 ‘low volume’ facilities in the bid to accelerate progress towards the UNAIDS 90–90-90 targets as visually represented in Fig. 1. Furthermore, our study underscores the importance of increasing domestic financial responsibility for the national HIV response in Uganda [45]. We call for the fast tracking of the proposed AIDS Trust Fund (ATF) to be financed through a levy on soft drinks by Ugandan consumers in the quest to increase local ownership of the national HIV response [46–49].

### Limitations

Our study had several limitations. A case study design such as that presented here is not intended to be broadly transferable to other settings: our aim was to understand the effects of transition from the perspective of patients and HIV service managers in a range of different contexts. The GP policy in Uganda was implemented in a dynamic context. For example, it was a challenge to untangle the precise effects of donor transition on HIV service delivery in Uganda due to other on-going processes and contextual factors. For instance, for much of 2017, due to a short fall in government health sector funding, Uganda experienced prolonged stock-outs of antiretroviral medicines country-wide which coincided with data collection [50], and were not limited to transition facilities. Additionally, in Uganda, PEPFAR’s implementation of the ‘geographic prioritization’ policy happened at the same time as the rationalization of PEPFAR support across geographic sub-regions and multiple other PEPFAR policies which might have had interactions in ways in which our study could not tease out.

## Conclusion

Participants perceived transition to have had the effect of narrowing the scope and quality of HIV services offered by case-study facilities due to a reduction in HIV funding as well as the loss of the additional personnel previously hired by the PEPFAR implementing organizations for HIV programming. Our study suggests that the effects of donor transition were compounded by contextual factors in our study sites such as broader governance deficits in newly created districts and the resource-limited operational context of transitioned health facilities. More research focused on patient and provider perspectives in response to donor transition are necessary to facilitate future transition processes, as well as determination of post-transition support. Additionally, further insights into how donor transition changes the services offered to patients and the extent to which patients and providers are involved in these decisions would be helpful.

## Supplementary Information

**Additional file 1: Supplementary file 1.** IDI Guide with facility in-charges.

**Additional file 2: Supplementary file 2.** FGD guide with patients.

## Data Availability

The datasets generated during and/or analyzed during the current study are not publicly available due to ethical reasons but are available from the corresponding author on reasonable request.

## Abbreviations

AIDS: Acquired Immune Deficiency Syndrome
ART: Anti-retroviral therapy
ARVs: Anti-retrovirals
GP: Geographic Prioritization
MOH: Ministry of Health
PEPFAR: The Presidents’ Emergency Plan for AIDS Relief
RA: Research Assistant
SSA: Sub-Saharan Africa
WHO: World Health Organization
