# Supplementary material for: The impact of loss of PEPFAR support on HIV services at health facilities in low-burden districts in Uganda
Source: BMC Health Serv Res. 2021 Apr 1;21:302. doi: 10.1186/s12913-021-06316-4 (PMC8017884; doi:10.1186/s12913-021-06316-4)
Supplement: Supplementary file 1 — Additional file 1: Supplementary file 1. IDI Guide with facility in-charges. [file 12913_2021_6316_MOESM1_ESM.docx]

Annex 1: Project SOAR – Longitudinal Case Studies of PEPFAR Geographic Prioritization

Semi-Structured Interview Guide – Facility In-Charge

# Introduction

Thank you for agreeing to meet us.

We are conducting an assessment of PEPFAR’s geographic prioritization process; that is, the process through which sites have transitioned from PEPFAR support to central support. We are interested in the processes that took place before transition to prepare and after transition under central support. Our goal is to provide practical information to local and national government, PEPFAR and other partners about how the transition process took place and whether it has affected how services are delivered. As part of the overall evaluation, we are conducting case studies with a number of specific facilities.

| Name of Organization |  |
| --- | --- |
| Your name |  |
| Designation |  |
| Work Area |  |
| Postal address |  |
| Telephone |  |
| E-mail address |  |

**OBTAIN INFORMED CONSENT**

*NOTE TO INTERVIEWER: This is a guide to the interview. You should cover* ***all the main numbered questions*** *in this interview form. You should use the probes selectively, according to the type of knowledge that the respondent conveys, and what you have already found out from documents and other interviews.*

# Interview Questions

1. Can you tell me a little about your current role, and how familiar you are with this facility?

*INTERVIEWER: If the respondent does not seem at all familiar with the case study facility, then please ask if there is someone else who is more familiar with the facility whom you could talk to.*

1. Are you familiar with the recent transition from [PEPFAR OR IMPLEMENTING PARTNER] support to government?

*PROBE:* Have you had any recent changes in how [IMPLEMENTING PARTNER] has supported the facility?

- 1. Were you involved at all in this transition process at this facility?

*INTERVIEWER: If the respondent does not seem at all familiar with transition, then please ask if there is someone else who is more familiar with transition whom you could talk to.*

1. Can you tell me about what types of support this facility was receiving from [IMPLEMENTING PARTNER] before transition?
   1. Examples: staff hiring and salaries, commodities, training, funding, support for reporting, patient incentives, etc.
   2. Any support for non-HIV services, like maternal and child health? E.g. antenatal care, immunizations, malaria, etc.
2. Can you explain to me why this facility was selected to lose support from [IMPLEMENTING PARTNER]?
   1. When did you find out that the facility would be transitioning?
   2. Who explained the process to you and your colleagues?
   3. How were facility staff members informed about the transfer process?
   4. How were patients informed about the transfer process?
3. What support was provided to this facility in order to prepare it for the transition process?
   1. Were there specific activities that were done to prepare?
   2. Were there any assessments done prior to transition to determine facility needs, and if so what were their findings? *(NOTE TO INTERVIEWER: ask for copies of the assessment if possible)*
      1. Who conducted it?
      2. When?
      3. How was it scheduled?
      4. How were the assessment results shared with the facility or with local government?
   3. Were meetings held between the facility, [IMPLEMENTING PARTNER] and government?
      1. When? How many?
      2. What was discussed?
   4. How did you involve facility staff and patients in planning for the transition?
   5. Which staff, if any, was most affected by the transition?
   6. Who provided most of the support to get the facility ready for transition?
4. Can you tell me a little about the actual transfer process, and how this went?
   1. Was there any confusion in terms of the transition and how it would be implemented? Please explain.
   2. Were all supported activities transferred at the same time?
5. How are facility services supported now after transition?
   1. What kind of support has the facility received from government? E.g. [NATIONAL AIDS CONTROL ORGANIZATIONS], central MOH, county/district health offices, medical bureaus (Uganda), etc.
   2. What kind of support has the facility continued to receive from the [IMPLEMENTING PARTNER], if any?
   3. Did the facility receive any funding from [IMPLEMENTING PARTNER] after transition? If so, do you know what this covered?
   4. What will this support look like in the next year? Longer-term?
6. What happened to the facility immediately post-transition?
   1. What changes did the facility have to make in the way it operated after transition? E.g. changes to reporting, staffing, etc.
7. How have clinical services at the facility changed post-transition?
   1. Clinical changes:
      1. HIV clinical services: HIV testing, treatment, referrals
      2. Community outreach
      3. Pediatric services
      4. Non-HIV services: antenatal care, family planning, malaria, tuberculosis
   2. Why have these changes taken place?

*PROBE:* changes related to transition or other contextual issue?

- 1. Did you anticipate any of these changes?
  2. Are there any plans to address these changes?

1. How has the management and organization of the facility changed post-transition?
   1. Health systems changes:
      1. Health workforce
      2. Commodity supply
      3. Budgets
      4. Reporting to DHIS
      5. Infrastructure
      6. Any difference between HIV and non-HIV services?
   2. Any changes to user fees? If so, for what services?
   3. Why have these changes taken place?

*PROBE:* changes related to transition or other contextual issue?

- 1. Do you see these as changes for the better or the worse?
  2. Did you anticipate any of these changes?
  3. Are there any plans to address these changes?

1. How has the facility performed over the longer term since transfer?
   1. Has the facility been able to adopt new practices, like test and treat?
      1. If so, was this easy or difficult to do?
      2. If not, why not? Any plans to do this in the future?
   2. How has the transition affected turnover of staff post transfer?
   3. How has the transition affected staff motivation or performance?
2. How has the transition affected service coverage?
   1. Why has this happened?
   2. Have there been effects on all the HIV services offered? E.g. PMTCT, ART, prevention, testing, etc.
      1. Why or why not?
   3. How has transition affected non-HIV services, like maternal and child health?
      1. Why has this happened?
3. How has the county/district health system responded to the transfer of these facilities away from PEPFAR support? Please explain.
   1. How has your relationship with the county/district health office changed?
   2. Have you collaborated with other facilities? E.g. referrals, commodities, staff.
4. In your view what else should have been done prior to the transfer in order to help with the transition process, which was not done?
5. Is there anything else significant about how the transition process has occurred at this facility that we should know about?

**Thank you for your time and contribution**
