# Supplementary material for: The impact of loss of PEPFAR support on HIV services at health facilities in low-burden districts in Uganda
Source: BMC Health Serv Res. 2021 Apr 1;21:302. doi: 10.1186/s12913-021-06316-4 (PMC8017884; doi:10.1186/s12913-021-06316-4)
Supplement: Supplementary file 2 — Additional file 2: Supplementary file 2. FGD guide with patients. [file 12913_2021_6316_MOESM2_ESM.docx]

Project SOAR – Longitudinal Case Studies of PEPFAR Geographic Prioritization

Focus Group Guide – Patients (ROUND 2)

# Introduction

Thank you for agreeing to meet us.

We are studying the transfer process of PEPFAR support to facilities to the local government, so that we can provide practical information to local and national government, PEPFAR and other partners about how the transition process took place and whether it has affected how services are delivered. We conducted a first round of interviews earlier this year, and we have returned to understand how things have changed since then.

| Name of facility | --------------------------------------------------------------------------------- |
| --- | --- |
| Names of community members | ---------------------------------------------------------------------------------  ---------------------------------------------------------------------------------  ---------------------------------------------------------------------------------  --------------------------------------------------------------------------------- |
| Postal address of the facility | ---------------------------------------------------------------------------------  ---------------------------------------------------------------------------------  ------------------------------------------------------------------------------------------------------------------------------------------------------------------ |
| Telephone of facility | --------------------------------------------------------------------------------- |
| E-mail address of facility | --------------------------------------------------------------------------------- |

**OBTAIN INFORMED CONSENT**

*NOTE TO INTERVIEWER: Please cover* ***all the main numbered questions*** *in the interview guide. Probes should be used selectively based on the kind of knowledge that the respondents convey and what you have already found from documents relating to transition and other interviews.*

# questions

1. Please can you introduce yourself and tell me how long have you been attending this facility?

*INTERVIEWER: Please ask each individual their name, age and how far they live from the facility.*

1. Are you aware that this facility has recently shifted away from receiving support from [PEPFAR/IMPLEMENTING PARTNER]?
2. What different organizations support this facility now?
   1. What kinds of support do they provide for patients like you? E.g. refreshments, support to meetings, family support
   2. Have these changed at all since May 2017? E.g. new organizations started, old organizations left, other changes.
3. What changes to HIV services have you noticed since May 2017?
   1. Was there any interruption in services?
   2. Do you see any changes in the types of services that the facility provides? If so, please explain.

*PROBE*: treatment, prevention, outreach.

- 1. Do you see any changes in the quality of services that the facility provides? If so, please explain.
  2. Can you explain why these changes have taken place?
  3. Have there been any changes to the drugs that are available at this facility?
  4. Have there been any changes in the types of laboratory tests that are available? the turnaround on obtaining test results changed?
  5. How have costs at this facility changed? E.g. For services, drugs or tests.

1. What changes to non-HIV services, like maternal and child health, have you noticed since May 2017?
   1. Was there any interruption in services?
   2. Do you see any changes in the types of services that the facility provides? If so, please explain.

*PROBE*: ANC, immunizations, etc.

- 1. Do you see any changes in the quality of services that the facility provides? If so, please explain.
  2. Can you explain why these changes have taken place?
  3. Have there been any changes to the drugs that are available at this facility?
  4. Have there been any changes in the types of laboratory tests that are available? the turnaround on obtaining test results changed?
  5. How have costs at this facility changed? E.g. For services, drugs or tests.

1. Since May 2017, have there been changes to the way that services are delivered at this facility? E.g. changes in staffing, the way services were provided etc.
   1. Did the facility makes any changes in the way it operates?
   2. Do you think the staff are as motivated as they were before?
   3. Has there been much turnover of staff?
2. How do you feel about the changes at the facility recently?
   1. How have you coped with these changes?
      1. How have other people you know coped with these changes?
   2. Would you consider switching to another facility to receive your services? Why/why not?
      1. If so, where would you go? Why?
      2. If so, do you think it would be harder or easier to get the care you want? Why/why not?
3. Do you think there is anything else significant about changes at this facility that we should know about?

***Many thanks for your help and time.***
